# Ghana’s path towards eliminating lymphatic filariasis

**DOI:** 10.1186/s41182-024-00596-2

**Published:** 2024-05-11

**Authors:** Munawar Harun Koray

**Affiliations:** Upper West Regional Health Directorate, Wa, Ghana

**Keywords:** Lymphatic filariasis, Ghana, China, Elephantiasis, Elimination

## Abstract

Lymphatic filariasis, also known as elephantiasis, is a debilitating parasitic disease that has been prevalent in various parts of the world, including China and Ghana. This paper explores the historical context of lymphatic filariasis in Ghana and China, as well as the fights towards eliminating the disease in both countries. The review also covered the strategies employed by the Chinese government to eliminate lymphatic filariasis and the key lessons that Ghana can learn from China's success. The discussion highlights the importance of political commitment, multisectoral collaboration, tailoring control strategies to local contexts, adopting a comprehensive approach, and emphasising health education and community mobilisation. By adopting these lessons and fostering a robust national strategy, engaging diverse stakeholders, and ensuring active community involvement, Ghana can work towards achieving lymphatic filariasis elimination, improving public health, and fostering sustainable development.

## Background

Lymphatic filariasis (LF), also known as elephantiasis, is a leading neglected tropical disease (NTD) caused by three parasitic worms: *Wuchereria bancrofti, Brugia malayi, and B. timori* and transmitted by five genera of mosquitoes, namely *Culex, Anopheles, Aedes, Mansonia, and Ochlerotatus* [1–4]. LF is particularly transmitted by *Culex quinquefasciatus, Anopheles gambiae s.l.* and *Anopheles funestus* especially in sub-Saharan Africa [1, 5].

Globally, LF has been identified as one of the causes of physical disability, mental illness, social and financial losses contributing to stigma and poverty [6–8]. Traditionally, LF has been prevalent in regions such as the Americas, the Pacific, Africa, and Asia [9]. An estimated 1 billion individuals across 72 nations were susceptible to LF infection, with a minimum of 36 million people experiencing the associated morbidities since the initiation of the World Health Organization's (WHO) Global Programme to Eliminate Lymphatic Filariasis (GPELF) [10].

In 1993, recognizing advancements in LF detection and treatment, the International Task Force for Disease Eradication listed LF, alongside diseases like cysticercosis and polio, as highly eradicable. Following the 1997 World Health Assembly (WHA) Resolution WHA 50.29, which called for LF eradication efforts, the GPELF was launched in 2000 with a goal to eliminate LF by 2020 [11]. Its strategies included halting LF transmission via mass drug administration (MDA) to at-risk populations and managing LF's health impacts by providing a basic care package [13–15].

Two decades into the GPELF, numerous countries have eradicated LF nationally, delivering an estimated 8.6 billion treatments since its  inception in the year 2000 to curb LF infection [6]. By 2018, global LF infection dropped to 51 million, a 74% decline, with around 692 million people no longer requiring preventive treatment [6]. Despite these achievements, some countries, particularly in Sub-Saharan Africa like Ghana, struggle to meet GPELF's objectives [12–14]. In response, GPELF has outlined new targets in the 2020 NTD Road Map for further LF elimination efforts [6, 15].

The GPELF indicated  that a total of 58 LF endemic nations, accounting for 80% of the total endemic countries, have fulfilled the criteria for confirming the eradication of LF as a disease of public health concern by 2030. These countries have maintained infection rates below the target levels for a minimum of 4 years after ceasing mass drug administration (MDA). They have also ensured the provision of the necessary care package in all regions with identified patients. In addition, 72 endemic nations (100%) engage in surveillance following MDA or validation. The last aim being that there has been a reduction of LF prevalence to zero in an entire population requiring the halting of MDA.

For Ghana to achieve these new set aims of GPELF, pragmatic steps must be taken and lessons adopted from countries, like China, that have successfully eliminated LF. The purpose of this review is to present the LF situation in Ghana; the epidemiology, and control measures taken, as well as the successes achieved so far. The review will also cover the challenges barring the successful elimination of LF in Ghana. The review will present the success story of China in its journey towards eliminating of LF. Lessons based on the Chinese story will be presented to facilitate Ghana’s effort to achieving the LF elimination by 2030.

## Methods

A comprehensive electronic search across various databases, such as PubMed, CINAHL, MEDLINE, EBSCO, and Google Scholar, alongside specific journals, such as the Ghana Medical Association Journal and African Journals Online (AJOL), was conducted to find literature on LF in Ghana and China. Using search terms such as “lymphatic filariasis in Ghana” and “China elimination of lymphatic filariasis”, connected by Boolean operators, and following up on references from articles, resulted in 61 articles and abstracts. Of these, 38 met the inclusion criteria focusing on the epidemiology, burden, control, management, and elimination programmes of LF in Ghana and China, without time restrictions and published only in the English.

Figures and tables were reproduced in this review as published in the original articles, taking into consideration the terms of the Creative Commons Attribution 4.0 International License (http://creativecommons.org/licenses/by/4.0/), under which the articles were distributed.

## Findings

### Lymphatic filariasis situation in Ghana and China

**Fig. 1:**
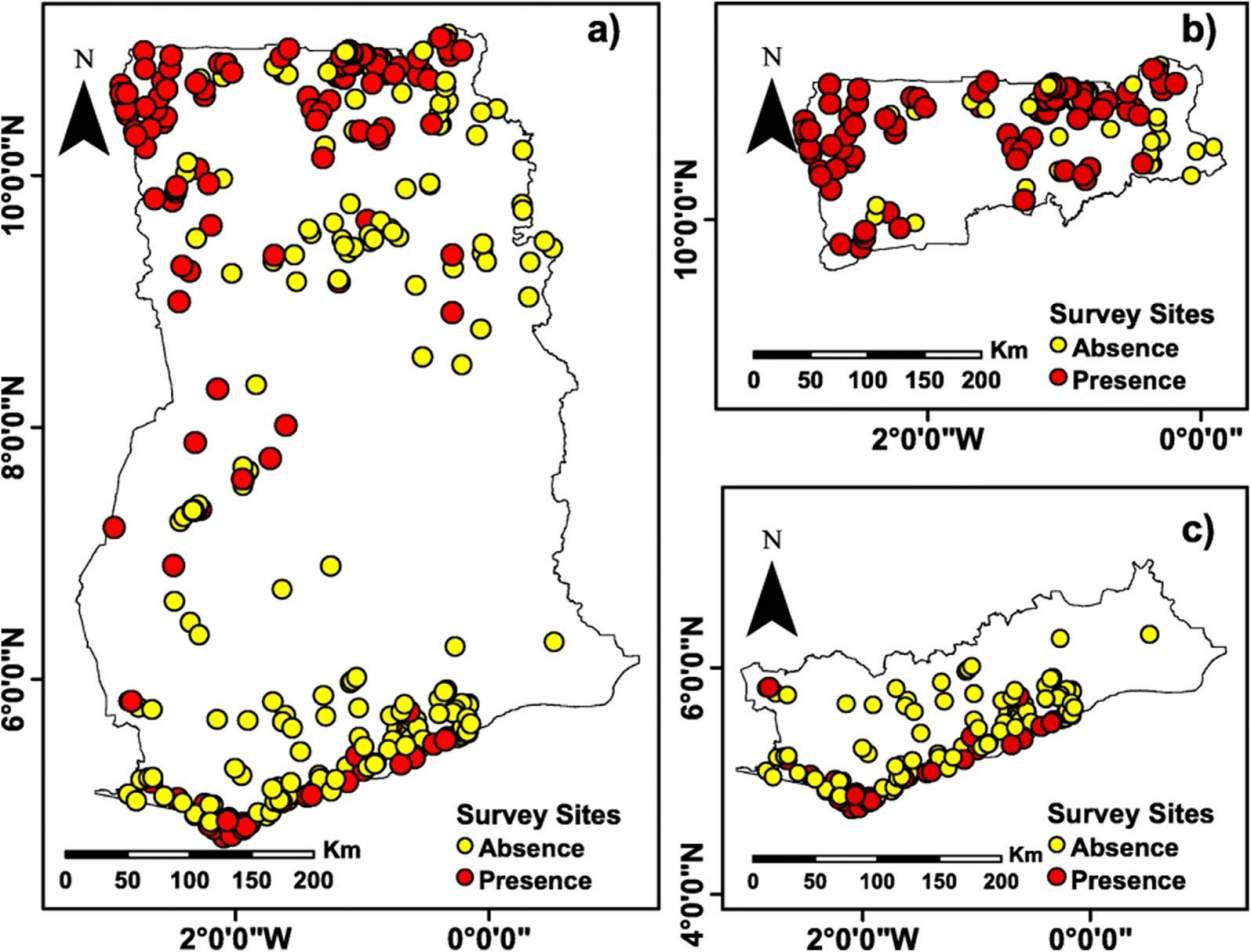
mfa cases in communities surveyed from 2000 to 2014 (The yellow dots represent absence and red shows presence of mfa), **a** Country Wide, **b** Northern Zone and **c** Southern Zones (Source: Kwarteng et al. [14])

The case of LF in Ghana, like in any other endemic country, is a huge public health concern to both healthcare managers and the population due to the psychosocial consequences and debilitating effect of the condition. In addition, LF poses a socio-economic implication for the afflicted individuals, their families, the healthcare system and the nation at large. Research on LF has demonstrated variations in the occurrence and range of symptoms across two separate geographic areas of Ghana; Northern regions and the Southern regions [16, 17]. An examination of mf cases across 430 communities in Ghana revealed that LF infection was predominantly located in the northern and southern areas, and also certain sections of the country's middle belt [14]. As observed by the red dots in Fig. 1, Microfilariae (MFA), the larva stage of parasitic worm responsible for causing filariasis, was present along the coastal communities of southern Ghana, specifically, the Western and Central Regions. In contrast, the northern part of the country experienced a more extensive spread of mf cases across various districts, with a considerably higher incidence rate compared to that of southern Ghana [14].

Microfilaremia (11.3%), breast lymphedema (6.6%), hydrocele (20.3%), and elephantiasis (1.7%) are significantly more prevalent in the northern region of Ghana than in the southern region (0.6%, 6.1%, 5.2%, and 0.7%, respectively) [14, 18]. In much earlier national surveys, the prevalence of microfilaraemia ranged from 0 to 20% by region [17]. In the Kassena Nankana district of the Upper East region, a highly endemic district, the prevalence of hydrocele was 30.8%, and elephantiasis of the leg was 3.8% in the population aged 10 years and older [19, 20].

Although the fundamental causes of the disparities in the spread of LF are unknown, they may be due to the presence of different parasitic strains in the country, particularly *Wuchereria bancrofti* [18, 21]. In addition, environmental and weather factors, including altitudes exceeding 200 m, average daily rainfall from 2.6 to 3.8 mm, and average daily temperature fluctuations from 24.5 to 26.0°C, impact the distribution of *Anopheles gambiae*, a vector involved in transmitting LF in Ghana [22] (Fig. 1).

In China, lymphatic filariasis (LF), has been recognized in records dating back to 600–700 B.C., suggesting diseases with LF-like symptoms [23]. Notably, during the Sui dynasty, physician Chao Yuanfang described diseases in his writings that mirrored LF's symptoms, such as hydrocele and lymphedema [24]. Prior to control efforts, China faced widespread LF caused by *Wuchereria bancrofti* and *Brugia malayi*, especially in its central and southeastern regions [24, 25]. By the 1980s, an estimated 31 million people were affected, with 22 million cases due to bancroftian filariasis and 9 million to *Malayan filariasis*, spanning across 864 counties in 16 provinces/regions [24]. Unique to the epidemiology in China, *W. bancrofti* and *B. malayi* lacked reservoir hosts, and transmission was influenced by the geographical context of temperate and subtropical areas [26]. The vectors showed varied natural infection rates, notably low overall, with the highest recorded infective larva rate at 6.8% in 1968 among *An. Sinensis* mosquitoes [24].

### Filariasis elimination programmes in Ghana and China

Both Ghana and China adopted strategies which were in line with WHO recommendations, which focuses on the use of MDA, surveillance and monitoring to reduce LF transmission and with strong WHO collaboration. However, the scale and scope of the MDA differed, as well as the implementation strategies, the geographical and epidemiological context and the outcome and progress.

**Fig. 2:**
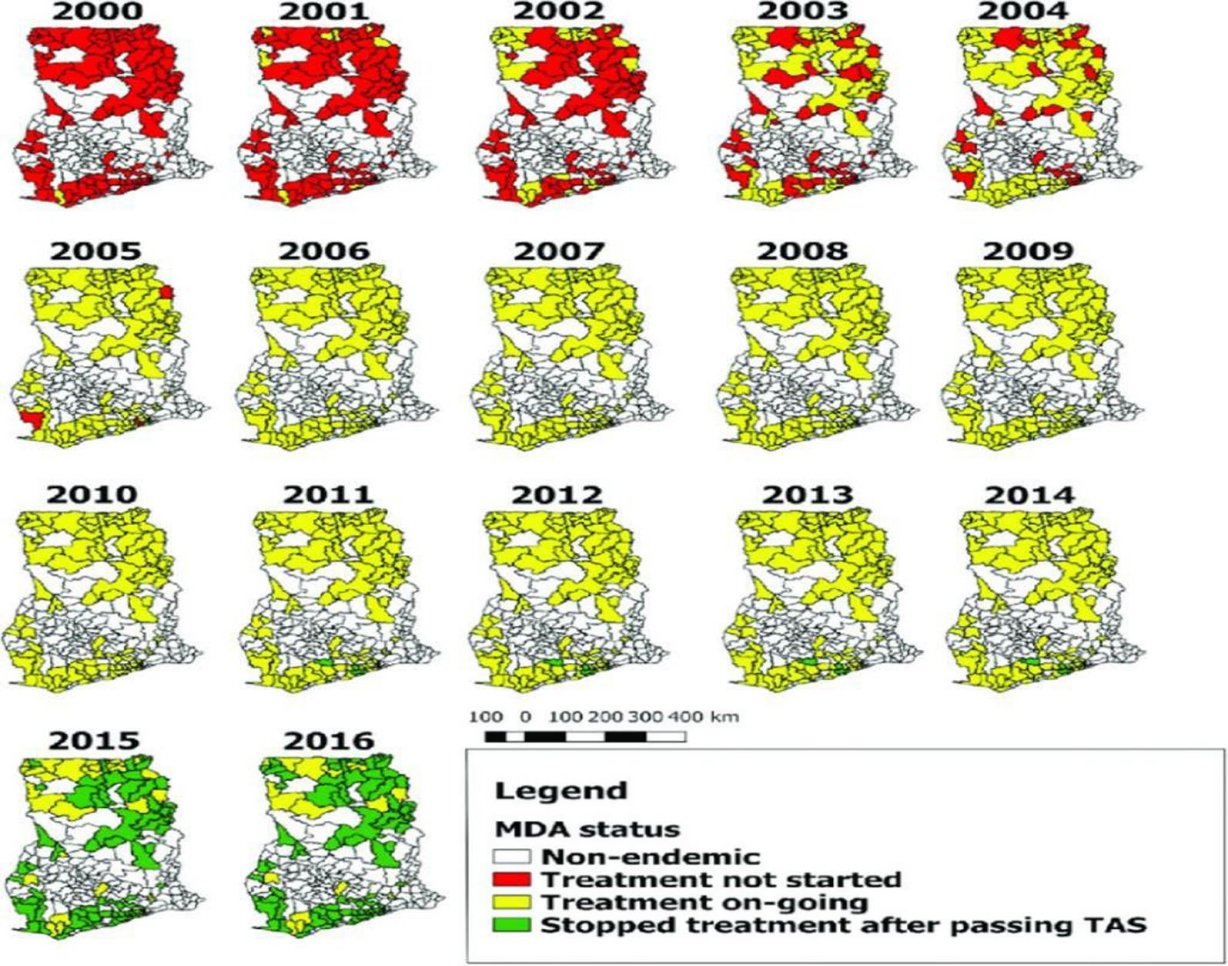
MDA implementation in Ghana by districts from 2000 to 2016 (Source: Biritwum et al. [27])

In Ghana, the Filariasis Elimination Programme (GFEP), now part of the Ghana’s Neglected Tropical Disease Programme, was founded in June 2000 to achieve the GPELF goal of eliminating LF globally. Ghana initially had 49 LF-endemic districts out of 110, which increased to98 of 216 districts (45%) after district re-demarcation [27]. The GFEP administered an annual MDA of 150 μg/kg ivermectin and 400 mg albendazole through community-directed treatment for countries co-endemic with LF and onchocerciasis [28, 29]. This initiative started in 10 districts and expanding annually from 2000 to 2006 [21, 27, 30] (Fig. 2). MDA was administered in implementation units (IU) with antigen prevalence greater than 1%, targeting individual over 5 years and older, except pregnant women, nursing mothers, and ill people with the treatment lasting for 1–2 weeks.

In addition, GFEP conducted parasitological surveys and transmission assessment surveys (TAS) to monitor LF transmission, using antigenaemia prevalence in 6–7 years as an indicator [29, 31, 32]. Biritwum et al. (2019) described details of the TAS in Ghana [27]. TAS targeted a demographic different from that of the in-depth mf survey. In-response the various endemic districts across Ghana enrolled the MDA elimination programme. The surveys helped determine the effectiveness of MDA and guided the continuation or cessation of treatment efforts. TAS-qualified evaluation units (EUs) were closely monitored, and MDA was paused when TAS-1 success was achieved. Before LF is eliminated as a public health issue, TAS-2 and TAS-3 are undertaken after 2–3 and 5 years, respectively (Fig. 2).

**Fig. 3:**
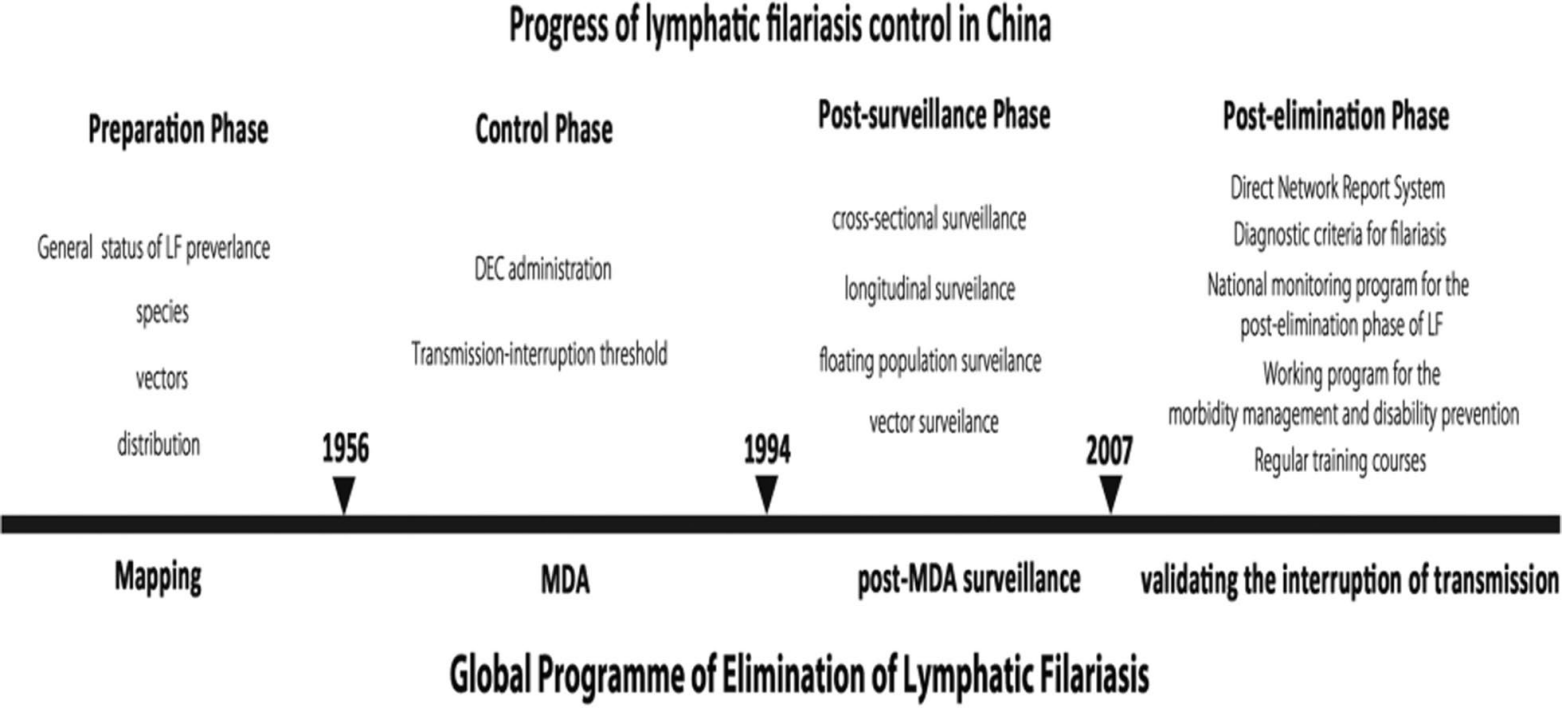
China's phases of LF elimination and GPELF stages towards LF elimination (Source: Fang and Zhang [3])

However, China adopted four phases in the journey of eliminating LF. These phases included the preparation phase, the control phase, the surveillance phase and the last phase being evaluation of transmission interruption.  These phases corresponds with the GPELF’s Mapping, MDA and post-MDA phases, including the validation of transmission interruption [29] (Fig. 3).

Three key strategies were employed in the control of LF in China: vector control, elimination of the sources of infection and the integration of vector control with elimination of infection sources. Diethylcarbamazine (DEC) emerged as the primary antifilarial drug after extensive trials, underpinning LF eradication efforts through three strategies: regular blood surveys and treatment, targeted treatment of microfilaremia cases alongside mass drug administration (MDA) in endemic regions, and integrating DEC salt for broader coverage [24]. The treatment scheduled is presented in Table 1. DEC salt's main benefit is its minimal and mild side effects, typically not requiring special treatments for patients. In addition, two to three blood surveys and therapies were recommended.

**Table 1:** Three DEC treatment used in China (Source: Fang and Zhang [3])

Regimen	Endemic extent	Target population	Dose
Selective treatment	Hypro-bancroftian filariasis	Microfilaraemia positive	3.0 g DEC over 3–5 days 4.2 g DEC over 7 days
	Hypro- and meso-malayan filariasis		1.5–2.0 g over 2–3 days
Mass drug administration (usually combined with selective treatment)	Hypro- and meso-bancroftian filariasis	> 5 years microfilaraemia-negative	3.0 g over 3–5 days
	Meso- and Hypro-malayan filariasis		1.0–2.0 g over 2–3 days
DEC-fortified salt	Meso- and Hypro-bancroftian filariasis	Whole population	50 mg DEC/day/person 6 consecutive months (9.0 g in total)
	Hypro-Malayan filariasis		50 mg DEC/day/person over 3–4 consecutive months (4.5–6.0 g in total)

Key among the strategies taken by the Chinese government to eliminate LF was a strong political will and sense of leadership [24]. Various institutions which were tasked in the elimination of LF were strengthened to carry out their responsibilities in a timely, effective and efficient manner. There was also a strong collaboration and cooperation between Chinese government departments and an active participation of individuals and communities in LF endemic areas [3]. International collaboration also played a significant role in eliminating LF in China. China worked closely with international organizations like the WHO to implement LF control measures and ensure that their efforts aligned with global best practices [25, 33]. There is a continuous and vibrant surveillance and monitoring to ensure the continued success of their LF elimination effort. China implemented a rigorous surveillance and monitoring system, which included periodic surveys, sentinel site surveillance, and case reporting [33]. In addition, China established the Morbidity Management and Disability Prevention (MMDP) programs to provide care for individuals suffering from LF-related morbidity, including hydrocele surgeries and lymphedema management.

In March 2006, the Ministry of Health of China officially submitted a national report to WHO on the elimination of LF in China in the fourth meeting of the Global Alliance to Eliminate Lymphatic Filariasis held in Fiji [25]. China was officially declared as successfully achieved the elimination of LF as a public health problem in May 2007 by WHO [24].

## Ghana’s progress towards eliminating LF

**Fig. 4:**
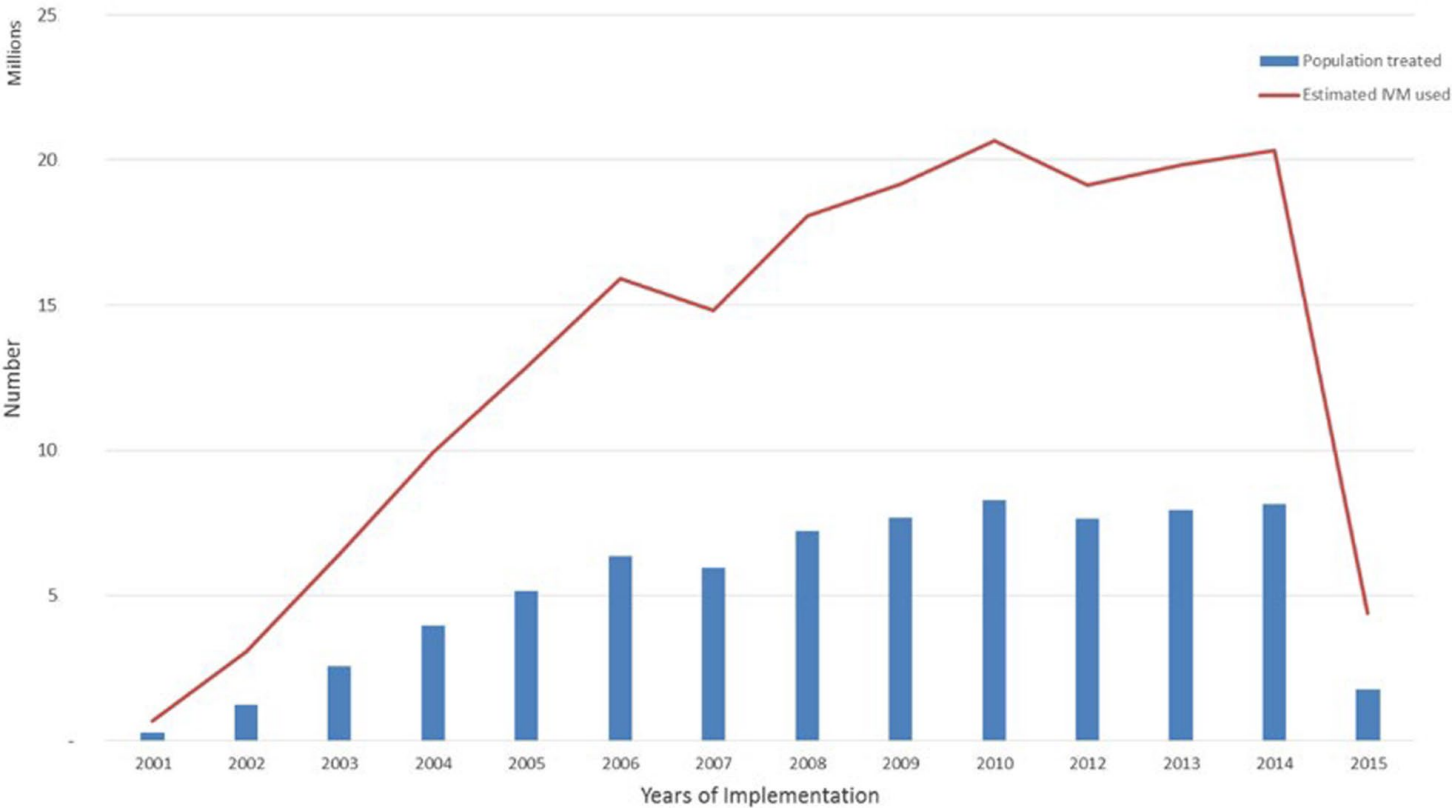
Population treated and ivermectin distribution from 2000 to 2015 (Source: Biritwum et al. [27])

China has successfully eliminated LF, however, Ghana is still making strides towards eliminating LF. Since the inception of GFEP in 2000, about 185 million ivermectin and 74 million albendazole treatments have been administered to over 74 million individuals in 98 endemic districts with significant scaling up efforts peaking in 2010 [27]. By 2015, 76 districts successfully passed TAS, ceasing MDA and highlighting the challenges of reaching all at-risk populations given that less than half of Ghana’s population resides in endemic areas (Fig. 4).

**Fig. 5:**
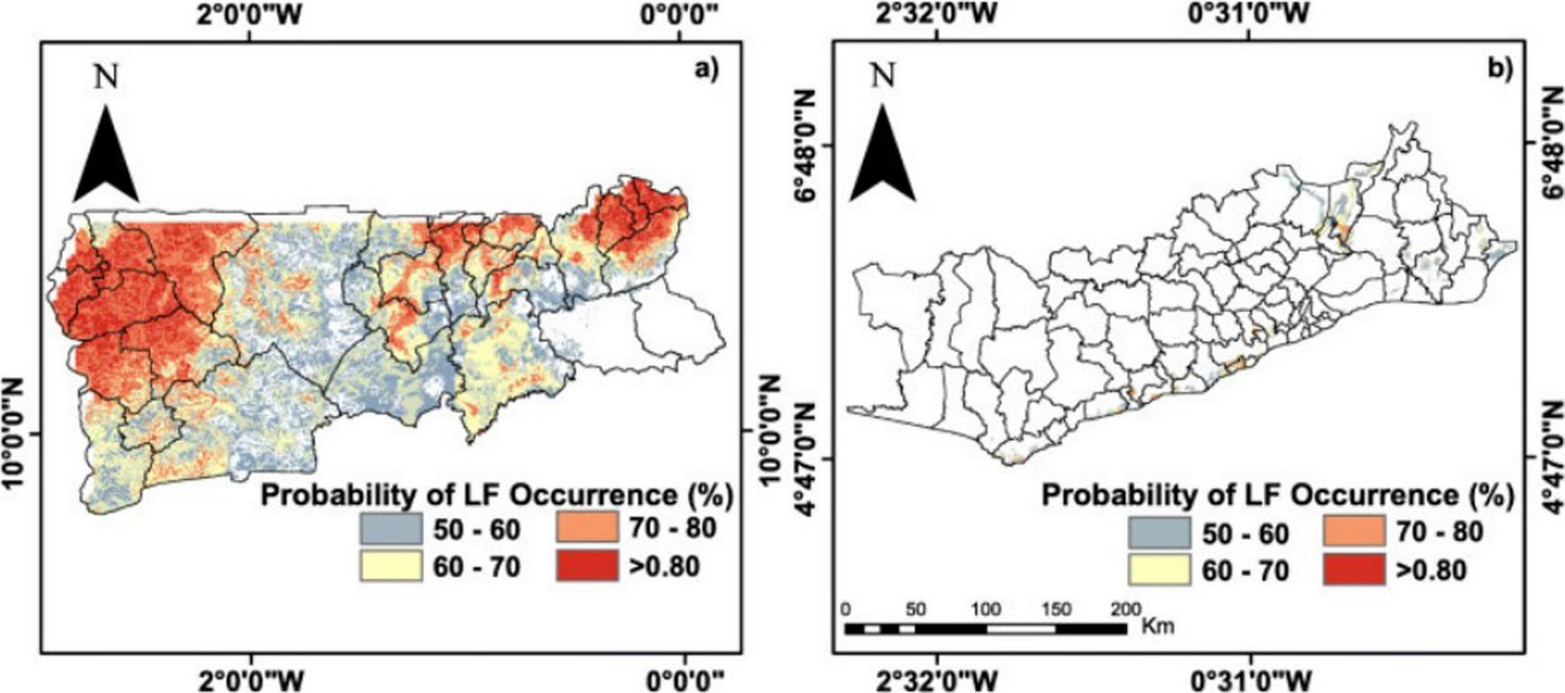
Maps showing the probability of LF occurrence in the northern and southern zones of Ghana. The red shades represent probability of ≥ 8 signifying most likely transmission area (Source: Kwarteng et al. [14] and de Souza et al. [18])

Initial data from 2000 revealed MF prevalence between 19.8% and 29.6% and ICT prevalence from 33.1% to 45.4% in districts selected for MDA (Ahanta West, Awutu-Efutu-Senya, Builsa, Kassena Nankana, and Sissala), leading to intensive surveillance and upscaling efforts [27]. Despite dramatic reductions in MF prevalence, certain districts with over seven MDA rounds still exceeded the 1% threshold for transmission interruption, complicating decisions on halting MDA due to inadequately designed TAS protocols [27]. From 2010 to 2015, TAS 1 surveys showed 76 districts with antigen prevalence below 1%, leading to the cessation of MDA in these areas [14]. By 2016, 82.7% of endemic areas had stopped MDA after up to 14 rounds, significantly reducing LF prevalence by 92% from 2000 to 2017 [34, 35]. However, despite widespread MDA and a substantial decrease in MF and ICT prevalence, some regions remain at high risk for LF, particularly in poor, rural communities of northern and southern Ghana, where environmental conditions favor transmission [14]. Areas still prone to LF due to geographical factors is highlighted in Fig. 5.

## Challenges in the eliminating of lymphatic filariasis in Ghana

Ghana began fighting lymphatic filariasis before WHO established GPELF in 2000 [16, 17, 19]. The GFEP increased the struggle against LF after its formation in June 2000. Since 2000, almost 98% of endemic districts and communities have received at least eight cycles of MDA. To meet the goal of GPELF in eliminating LF by 2020 [29], the programme has been monitored and evaluated to determine its successes and obstacles. Ghana, like other SSA nations, failed to meet this goal due to programme implementation challenges [36].

## Non-compliance to treatment

MDA implementation is hindered by community members' non-compliance to treatment. Dizziness, rashes, and general weakness scared some users away from the medicine [37, 38]. Most of them took the drugs from the volunteer but threw them away to avoid side effects [12]. Because they saw no bodily changes after taking the medicine, several people thought it had no health benefits. Others declined to join the MDA because they believed they were not at risk for LF infection, notably elephantiasis and hydrocele, due to the lack of clinical signs [35, 37]. Some community members believe medication are for treating and curing diseases, not as vaccinations. They believed that ivermectin is not recommended for healthy, pain-free people. Healthy people preferred immunizations over taking in drugs [34].

## Poor knowledge of the MDA

Poor MDA awareness keeps LF in the remaining endemic districts. Some community people were in the known of the MDA but not the importance of treatment for LF prevention [37]. Community members receive MDA information before drug distribution. However, the information solely focused on the drug distribution date and failed to educate community people regarding the importance of taking the treatment in eliminating LF, its potential side effects, and side effect management [39]. Many of the remaining endemic districts lack community engagement, which may have served as platform for education on MDA and its positive impact on reducing LF [35].

## Health system barriers

Weak supervision of volunteers by the district’s health management team (DHMT) is cited as one of the barriers preventing community members to accept the LF treatment [35, 39]. In addition, community members distrusted some volunteers. A section of the community members suspected that some MDA volunteers were political campaigners seeking political gains using the programme [37]. This made some community members decline the drugs. It is also reported by some community members that few volunteers were not committed to the work by ensuring the drugs are taken as prescribed [37]. Volunteers and health care workers attitudes hampered drug acceptance. Some believed some volunteers were unfriendly during drug delivery [37]. Healthcare professionals were unable to ensure medicine distribution procedures were followed [37].

## Time of implementation of MDA and surveys

Timing of the treatment distribution in communities, surveys, and ICT cards is another challenge [36, 39]. Most LF-endemic communities are rural and farming communities and the annual MDA often coincides with the season for planting. Thus, most community residents leave early for the farms and return late. Volunteers rarely meet household members before the move to their farms. Volunteers try to meet community members, but they often fall short of their MDA goal. Poor survey timing and ICT card quality caused technical issues [36]. These studies were done during the cold, sandy Harmattan season, usually December to February. This often result in dusty slides which contains blood stains and antigen tests. This prevented useful results and therefore the results were always discarded. Cold temperature causes vasoconstriction, making blood samples difficult to acquire, especially between 10 pm and 2 am. ICT cards stored overnight due to low visibility yielded many false positives. The ICT and MF blood slide results were unusable for programme assessment [36].

## Logistics and funding challenges

Every healthcare intervention depends on logistics and funding. LF elimination follows suit. Funding and logistics affected MDA upscaling and surveys in Ghana. Finance and logistics constraints in Ghana resulted in the modification of the WHO monitoring and supervision guideline [36]. Logistical issues prevented MDA in 2011 [27]. The GFEP budget and logistics dictated survey sample sizes and sentinel site numbers. This may affect programme decision-making and prevalence estimations. Volunteers and field officers may have been dissatisfied with the exercise due to funding issues [40]. Volunteers needed raincoats to distribute medications during the MDA, which generally takes place in the rain.

## Lessons from China’s successful elimination of lymphatic filariasis

Ghana can learn several valuable lessons from China’s success in controlling LF:

1. Political commitment: Strong political will and commitment from the government played a crucial role in China’s success [41]. Ghana can learn from this by ensuring that LF elimination remains a priority on the national health agenda, with dedicated resources and continuous support.

2. Multisectoral collaboration: China’s approach to controlling LF involved collaboration between various sectors, including health, education, and research institutions [33]. Ghana can benefit from fostering similar partnerships among stakeholders, such as government agencies, NGOs, and international organizations, to ensure a coordinated and effective response.

3. Tailoring control strategies to local contexts: China’s success can be partly attributed to the adaptation of control strategies to suit local conditions, including varying vector species, parasite strains, and socio-economic factors [3, 42]. Ghana can take a similar approach by developing context-specific strategies that address the unique challenges faced in various regions of the country.

4. Comprehensive approach: China employed a comprehensive strategy, encompassing mass drug administration, vector control, case detection, treatment, health education, and community mobilization [3, 24, 42]. Ghana can learn from this by ensuring that all aspects of LF control measures are addressed in a holistic manner, with equal emphasis on prevention, treatment, and management of complications.

5. Health education and community mobilization: Public awareness and community engagement were crucial components of China’s strategy [3, 42]. Ghana can benefit from intensifying health education efforts and actively involving communities in the planning and implementation of lymphatic filariasis control activities. This can help improve understanding of the disease, increase participation in MDA campaigns, and encourage early detection and treatment of cases.

## Conclusion

The successful elimination of LF in China serves as an inspiring example for countries like Ghana that is still grappling with this debilitating disease. By learning from China's experience and adopting key strategies such as political commitment, multisectoral collaboration, tailored local approaches, comprehensive control measures, and a strong emphasis on health education and community mobilization, Ghana has the ability to make significant strides towards eliminating LF by 2030. Ghana must develop and maintain a strong national strategy, engage diverse stakeholders, and ensure active community participation in the process. By following China's lead and implementing these strategies, Ghana can work towards eradicating LF as a public health issue, ultimately enhancing the health of its population and promoting sustainable development.

## Data Availability

The author of this review paper does not hold the right to the data used in this study, as it was obtained from existing published articles, of which are duly cited in this review.

## Abbreviations

DEC: Diethylcarbamazine
EU: Evaluation Unit
GFEP: Ghana Filariasis Elimination Programme
GHS: Ghana Health Service
GPELF: Global Programme to Eliminate Lymphatic Filariasis
L3: Third larval stage
LF: Lymphatic filariasis
IU: Implementation Unit
ICT: Immunochromatographic card test
MDA: Mass drug administration
MFA: Microfilariae
NTD: Neglected tropical disease
SSA: Sub-Saharan Africa
TAS: Transmission Assessment Survey
WHA: World Health Assembly
WHO: World Health Organization
